# T and B cell responses in different immunization scenarios for COVID-19: a narrative review

**DOI:** 10.3389/fimmu.2025.1535014

**Published:** 2025-03-18

**Authors:** Eva Piano Mortari, Francesca Ferrucci, Irini Zografaki, Rita Carsetti, Luciano Pacelli

**Affiliations:** ^1^ B Lymphocytes Unit, Bambino Gesù Children’s Hospital, istituto di ricovero e cura a carattere scientifico (IRCCS), Rome, Italy; ^2^ Medical Department, Covid Franchise, Pfizer s.r.l., Rome, Italy; ^3^ mRNA & Antivirals Medical & Scientific Affairs International Developed Markets, Pfizer, Athens, Greece; ^4^ Medical Department, Internal Medicine, Pfizer s.r.l., Rome, Italy

**Keywords:** COVID-19, vaccines, homologous, heterologous, hybrid, B-cell, T-cell

## Abstract

Vaccines against COVID-19 have high efficacy and low rates of adverse events. However, none of the available vaccines provide sterilizing immunity, and reinfections remain possible. This review aims to summarize the immunological responses elicited by different immunization strategies, examining the roles of homologous and heterologous vaccination and hybrid immunity. Homologous vaccination regimens exhibit considerable variation in immune responses depending on the vaccine platform, particularly concerning antibody titers, B cell activation, and T cell responses. mRNA vaccines, such as mRNA-1273 and BNT162b2, consistently generate higher and more durable levels of neutralizing antibodies and memory B cells compared to adenovirus-based vaccines like Ad26.COV2.S and ChAdOx1. The combination of two distinct vaccine platforms, each targeting different immune pathways, seems to be more effective in promoting long-lasting B cell responses and potent T cell responses. The high heterogeneity of the available studies, the different dosing schemes, the succession of new variants, and the subjects’ immunological background do not allow for a definitive conclusion. Overall, heterologous vaccination strategies, combining sequentially viral vector and mRNA may deliver a more balanced and robust humoral and cellular immune response compared to homologous regimens. Hybrid immunity, which arises from SARS-CoV-2 infection preceded or followed by vaccination produces markedly stronger immune responses than either vaccination or infection alone. The immune response to SARS-CoV-2 variants of concern varies depending on both the vaccine platform and prior infection status. Hybrid immunity leads to a broader antibody repertoire, providing enhanced neutralization of variants of concern. Heterologous vaccination and hybrid immunity may provide further opportunities to enhance immune responses, offering broader protection and greater durability of immunity. However, from all-cause mortality, symptomatic or severe COVID, and serious adverse events at present it is not possible to infer different effects between homologous and heterologous schemes. Next-generation vaccines could involve tweaks to these designs or changes to delivery mechanisms that might improve performance.

## 1 Introduction

The novel coronavirus Severe acute respiratory syndrome coronavirus 2 (SARS-CoV-2) was first identified in 2020 during an outbreak of severe respiratory illness in Wuhan, China (1–3). The disease, called Coronavirus disease 2019 (COVID-19), rapidly spread globally, prompting the World Health Organization (WHO) to declare a Public Health Emergency of International Concern on January 30, 2020, followed by the declaration of a pandemic on March 11, 2020 (4). Over three years later, on May 5, 2023, WHO announced that while the pandemic persists, it no longer constitutes a global health emergency (5). By this time, most of the global population had developed immunity to the virus, either through vaccination (homologous or heterologous), infection, or a combination of both resulting in hybrid immunity.

Vaccines were developed at an unprecedented pace due to substantial financial investments, extensive scientific collaborations, and the expedited efforts of regulatory bodies. The first vaccine was approved 326 days after the sequencing of SARS-CoV-2 was completed (6). The Pfizer-BioNTech and Moderna mRNA vaccines received emergency use authorization in December 2020, followed two months later by the approval of the AstraZeneca adenovirus-based vaccine. In subsequent months, vaccines using diverse technologies, including viral vectors, protein subunits, and inactivated viruses were developed. All were licensed based on their high efficacy and low rates of adverse events (7–10), contributing to significant reductions in severe cases and fatalities from COVID-19, particularly in regions like Europe where mortality was reduced by up to 75% (11).

However, none of the available vaccines provide sterilizing immunity, and reinfections remain possible. Hybrid immunity, while offering improved protection, provides only 42% effectiveness against reinfection within 12 months (12). As the virus continues to circulate and mutate, new variants of concern (VOCs) emerge, exhibiting changes in transmission, infectivity, and disease severity (13). These variants may escape immune responses due to mutations in the receptor-binding domain (RBD) of the spike protein, further complicating public health efforts.

This review aims to summarize the immunological responses elicited by different immunization strategies, examining the roles of homologous and heterologous vaccination and hybrid immunity. Although different vaccines have been developed worldwide since the pandemic, this review will overview studies conducted with EMA/FDA approved vaccines that have been most extensively studied in clinical and preclinical setting. The goal is to determine whether sustained and effective immunity can be generated through improved vaccines, modified immunization strategies, or altered booster frequency and timing. The review will not evaluate vaccine effectiveness/efficacy from an “hard endpoint” perspective (e.g. hospitalization or death) but will rather deep dive into cellular and humoral response. The majority of the studies herein analyzed systemic humoral response (i.e. IgG), and therefore no inference can be made on specific Ig subclasses or tissue specific humoral immunity.

## 2 Overview of SARS-CoV-2 structure

The SARS-CoV-2 virion consists of 29 structural proteins, including spike (S), envelope (E), membrane (M), and nucleocapsid (N) proteins (14). These proteins play crucial roles in viral replication and interactions with host cells, making them prime targets for therapeutic interventions (14–17). The spike protein (S), responsible for viral entry, is composed of two subunits: S1 (which includes the RBD) and S2. The RBD facilitates binding to the human angiotensin-converting enzyme 2 (hACE2) receptor on host cells and initiates viral internalization (14). The major pathway of infection involves the proteolytic processing of the S protein by the transmembrane serine protease 2 (TMPRSS2), enabling virus host membrane fusion and release of the viral RNA in the cytosol (14). If the target cell does not express TMPRSS2, the virus enters via clathrin-mediated endocytosis and cathepsin L activates the fusogenic properties of S leading to the uncoating of the viral genetic material (14, 18). After infection, the virus remodels the host endoplasmic reticulum to build a specialized organelle, the replication organelle (RO), where the virus replicates. The viral genome is then assembled with the viral structural proteins to generate virions, which exit via vesicular carriers (19).

Vaccines that induce antibodies targeting the spike protein and RBD aim to block viral attachment and entry into host cells, thus preventing infection. As a result, the spike protein has been the major focus of COVID-19 vaccine development.

## 3 Approved vaccines

Several vaccines have been developed and approved by the European Medicines Agency (EMA; **Table 1**). Although their efficacy varies, all offer strong protection against severe disease, hospitalization, and death. The vaccines employ a range of technologies, including:

**Table 1:** Vaccines approved for COVID-19 in the EU.

Vaccine SPC*	INN/common name	Date of first authorization / Latest renewal	Marketing authorization holder	Platform/type	Indication	Target variant/Update strain date
Bimervax (131)	Damlecovatein	30 March 2023	Hipra Human Health, S.L.U.	Recombinant spike protein receptor binding domain fusion heterodimer - SQBA adjuvant	Booster for active immunization to prevent COVID-19 in individuals 16 years of age and older who have previously received a mRNA COVID-19 vaccine	XBB.1.16 / 17 oct 2024 / NA
Nuvaxovid (132)	NVX-CoV2373	20 Dec 2021 / 3 Oct 2022	Novavax CZ, a.s.	Recombinant spike protein – Matrix-M adjuvated	Active immunization to prevent COVID-19 caused by SARS-CoV-2 in individuals 12 years of age and older	JN.1 / 07 Oct 2024
VidPrevtyn Beta (166)	COVID-19 vaccine (recombinant, adjuvanted)	10 Nov 2022	Sanofi Pasteur, France	SARS-CoV-2 spike protein - AS03 adjuvated	Booster for active immunisation to prevent COVID-19 in adults who have previously received an mRNA or adenoviral vector COVID-19 vaccine	B.1.351 strain / NA
Comirnaty (119)	Bretovameran, COVID-19 mRNA vaccine (nucleoside modified)	21 Dec 2020	BioNTech Manufacturing GmbH	mRNA encoding Spike protein	Active immunization to prevent COVID-19 caused by SARS-CoV-2 in individuals 8 months of age or older	JN.1/ 27 Jun 2024
Spikevax (120)	COVID-19 mRNA vaccine (nucleoside-modified)	6 Jan 2021 / 3 Oct 2022	Moderna Biotech Spain, S.L.	mRNA encoding Spike protein	Active immunization to prevent COVID-19 caused by SARS-CoV-2 in individuals 6 months of age and older	JN.1 / 9 Sep 2024
Vaxzevria§ (129)	COVID-19 Vaccine (ChAdOx1-S [recombinant])	29 Jan 2021	AstraZeneca AB	Adenovirus encoding Spike protein	Active immunization to prevent COVID-19 caused by SARS-CoV-2 in individuals 18 years of age and older	Wuhan Ancestral / NA
Jcovden§ (130)	COVID-19 vaccine (Ad26.COV2-S [recombinant])	11 March 2021	Janssen-Cilag International NV	Adenovirus type 26 encoding the SARS-CoV-2 spike glycoprotein	Active immunization to prevent COVID-19 caused by SARS-CoV-2 in individuals 18 years of age and older	Wuhan Ancestral / NA

*Summary of Product Characteristics.

^§^Vaxzevria, Jcovden and Vidprevtyn Beta marketing authorization has been withdrawn by the Marketing authorization holder in 2024 (167).

NA, not applicable.

For complete information on efficacy or immunobridging data please refer to the SmPC of the listed product.

1. mRNA vaccines: Comirnaty (Pfizer-BioNTech) and Spikevax (Moderna), which use lipid nanoparticles to deliver SARS-CoV-2 spike mRNA that encodes the protein inside cells.
2. Viral vector vaccines: Vaxzevria (AstraZeneca; marketing authorization withdrawn on 27/03/2024) and Jcovden (Johnson & Johnson; marketing authorization withdrawn at the request of the marketing authorization holder on 09/08/2024), using adenoviruses, respectively chimpanzee and human, to deliver genetic material encoding the spike protein.
3. Protein subunit vaccines: Nuvaxovid (Novavax) and Bimervax (HIPRA), which contain a recombinant SARS-CoV-2 spike protein, though they use different adjuvants. Another protein-based vaccine (Vidprevtyn Beta) has been authorized by EMA, but marketing authorization was withdrawn (Sanofi Pasteur; marketing authorization withdrawn on 11/03/2024).

All these vaccines can be used for primary series and booster doses, except Bimervax, which is recommended as a booster after mRNA vaccines and Vidprevtyn Beta (currently not authorized), which was recommended as a booster after mRNA or adenoviral vector COVID-19 vaccines.

## 4 B and T cell responses to natural infection

Following viral infection, the innate and adaptive immune systems work in concert to mount an effective response. Upon infection, the innate immune system is quickly activated to limit viral replication. Meanwhile, the adaptive immune system generates the most specific and effective tools of protection, high-affinity memory B cells and antibodies.

In the case of COVID-19, humans were exposed to a virus never seen before and adapted to the immune system of a different species. The extraordinary activation of the innate immune system led to severe systemic and organ damage caused by the cytokine storm (20). The importance of the innate response to SARS-CoV-2 is demonstrated by the identification of inborn error of innate immunity in individuals with severe clinical disease. In ~ 3–5% of patients, previously undiagnosed primary immunodeficiencies affecting the antiviral response were identified (21). In addition, in 10%-20% of cases, autoantibodies directed against anti-type 1 interferons neutralized natural defenses, thus explaining the severity of COVID-19 (21).

The adaptive immune system is also activated by the infection. Antigen-presenting cells (APCs), including dendritic cells and macrophages, capture viral particles, process them, and activate the adaptive immune system (22, 23). B-cell responses can be divided into canonical germinal center (GC) and noncanonical extrafollicular (EF) responses. The GC response produces high-affinity memory B cells (MBCs), plasma cells (PCs), and serum antibodies capable of mediating long-term protective immunity. In the GCs, activated B cells introduce somatic mutations in the immunoglobulin genes and undergo the selection mechanism that promotes survival and further development of high-affinity memory B and plasma cells. Follicular helper T cells play a fundamental role in the GC reaction and are indispensable for the generation of protective and long-lived immune memory. Two weeks are necessary for the completion of the GC response (24), which is a very long time when fighting a virus. The EF response has the role of protecting the organism in the first days of infection and generates an early antibody response with near germline-encoded B-cell receptors (25). The two processes together lead to the production of neutralizing antibodies (both from the EF and GC reactions), and the development of memory cells. The balance between these two processes depends on the type of stimulation and may influence the evolution of the disease.

Severe COVID-19 results in defective GC reactions with more pronounced EF responses. The aberrant production of TNF-α disrupts the GC structure with impaired recruitment of Bcl-6+ follicular helper T cells (26) and results in production of antibodies unable to mediate disease resolution and favoring, instead, the progression of the viral infection (27–33).

Although critically ill patients show EF-like responses shortly after infection and lack GC reactions, convalescent patients usually manage to develop a MBC response. Multiple studies tracking B-cell responses over time (3 to 8 months) have reported an increase of spike-specific MBCs, with the MBC population remaining relatively stable between six and 12 months after infection (22). After natural infection, memory B and T cells persist for months, providing durable protection against severe disease (22). While antibody titers naturally decline over time, MBCs increase in frequency (29, 31, 34, 35). MBCs play an important function upon a second encounter with the virus. Not only they rapidly differentiate into antibody-secreting cells (ASCs) and increase antibody concentration in the peripheral blood, but they can also modify their immunoglobulins and generate new MBCs and PCs with broader specificity (36). Remodeling of MBCs is driven by pre-existing high-affinity antibodies (37). Neutralizing antibodies control viral infection by inhibiting viral entry into the cells. Specific antibodies also facilitate viral clearance through phagocytosis, activation of the complement system, and addressing natural killer (NK) cells towards infected cells. Immunological memory and antibody levels are maintained by MBCs that persist in the lymphoid organs and by PCs, residing in bone marrow.

Following infection, T cellular immunity is also generated. CD4+ and CD8+ specific T cells can be detected seven days after infection and remain detectable for years (28, 35, 38–40). In individuals who recovered from SARS (caused by the SARS-CoV virus), T cells remain detectable 17 years after infection and cross-react with the novel coronavirus (41).

Thus, following SARS-CoV-2 infection, the coordinated efforts of the innate and adaptive immune systems are essential for an effective response. The innate immune system provides rapid defense but, in severe cases of COVID-19, its overactivation can lead to harmful inflammation and cytokine storm (42). The adaptive immune system, involving B and T cells, generates long-term protection through the production of high-affinity MBCs, neutralizing antibodies (43, 44) and T cells. While severe COVID-19 disrupts the GC response, leading to a reliance on early EF responses, convalescent patients also develop immunological memory (34, 45, 46). Memory B and T cells contribute to durable protection against severe disease (22, 47, 48).

## 5 Immunity after homologous vaccination

Homologous vaccination regimens show considerable variation in immune responses, largely dependent on the vaccine platform, particularly in terms of antibody titers, B cell activation, and T cell responses. The most accessible and efficient method to evaluate humoral immunity in response to COVID-19 vaccination is through the quantification of IgG antibodies specific to the spike protein or the RBD. This measurement, combined with the assessment of neutralizing activity, serves as a reliable indicator of the vaccine-induced immune response.

Both mRNA vaccines, mRNA-1273 (Moderna) and BNT162b2 (Pfizer-BioNTech), induce strong immune responses even after a single dose, with spike- and RBD-specific IgG antibodies reaching peak levels after the second dose (7, 8). Interestingly, both mRNA vaccines elicited antibodies targeting the spike protein (S1+S2) which also comprise the RBD, and these antibody levels remain elevated over time compared to adenoviral vector vaccines like Ad26.COV2.S (Johnson & Johnson) and ChAdOx1 (AstraZeneca), which mounted an antibody response targeting preferentially only the S1 subunit (49–51). mRNA vaccines consistently induce higher IgG and neutralizing antibody titers, which remain sustained for up to six months post-vaccination (52). In contrast, individuals who received Ad26.COV2.S or ChAdOx1 exhibit lower peak antibody responses, with a more rapid decline over time (52). Six months after vaccination, neutralizing antibodies were slightly lower in individuals who received BNT162b2 compared to mRNA-1273, but 100% of the recipients remained positive for spike-specific and RBD-specific IgG antibodies, as well as neutralizing antibodies (49). Although there is limited data on protein-based vaccines, NVX-CoV2373 (Novavax) was shown to generate neutralizing antibody titers comparable to mRNA vaccines (49). The decay rate of spike-specific IgG following NVX-CoV2373 was also similar to that of mRNA vaccines, suggesting comparable durability of the humoral immune response.

SARS-CoV-2 specific B cell responses can be evaluated using flow cytometry, measuring B cells that bind to spike and RBD fluorescent probes. All vaccines generate spike- and RBD-specific MBCs at 6 months post-immunization, with RBD-specific MBCs accounting for 15-20% of the spike-specific population (47, 53, 54). mRNA vaccines induce higher frequencies of specific MBCs compared to Ad26.COV2.S and NVX-CoV2373 at both 3.5 and 6 months post-vaccination (49). Notably, the kinetics of MBC responses differ from antibody responses, as MBCs continue to increase in frequency over time (49, 55), much like after natural infection. Turner et al. demonstrate that after the 2^nd^ dose of mRNA vaccination the GC reaction in the axillary lymph nodes was maintained for 12 weeks (56). This long-lasting GC reactions could explain why mRNA vaccines generate a higher proportion of activated MBCs, compared to Ad26.COV2.S and NVX-CoV2373 (57).

T cell responses are commonly evaluated through IFN-γ ELISpot, activation-induced marker (AIM) assays, or intracellular cytokine staining (ICS) (58). Both mRNA-1273 and BNT162b2 induce robust CD4+ and CD8+ T cell responses, peaking after the second dose and maintained for up to six months (59). mRNA-1273 produces slightly higher overall T cell responses than BNT162b2 (60), particularly for IFNγ+, TNFα+, and IL-2+ memory CD4+ T cells. In the case of CD8+ T cells, responses are dominated by IFNγ-producing cells, with many also expressing granzyme B, contributing to their cytotoxic capacity (61).

Both mRNA vaccines also stimulate spike-specific circulating follicular helper T (cTfh) cells, which play a critical role in supporting antibody production after vaccination (62–64) and have been demonstrated to persist six months after mRNA vaccination and showed a significant correlation with GC B-cell responses (64). The higher frequencies of Tfh could explain the higher concentration of antibodies generated by mRNA vaccines compared to adenovirus-based vaccines.

Adenovirus-based vaccines, such as Ad26.COV2.S, generate lower CD4+ T cell responses compared to mRNA vaccines (49, 65). These responses are characterized by less multifunctionality and lower levels of spike-specific memory CD4+ T cells. Although the induction of CD8+ T cells is also lower than that observed with mRNA vaccines, six months after immunization, the CD8+ T cell memory rates remain stable and are comparable to those seen in mRNA vaccine recipients (49). ChAdOx1 also induces lower CD4+ T cell responses compared to mRNA vaccines (49), but the CD8+ memory T cells generated in response to ChAdOx1 relocate to the respiratory tree to protect the mucosal tissue (66). The protein-based vaccine NVX-CoV2373 induces comparable CD4+ T cell frequencies to mRNA vaccines, with stable memory T cell levels at six months (49). However, NVX-CoV2373 generates lower frequencies of memory CD8+ T cells with minimal multifunctionality in a smaller fraction of vaccine recipients. Overall, all vaccines generate memory CD4+ T cells with both Th1 and Tfh populations, although mRNA vaccines and NVX-CoV2373 induce higher frequencies of spike-specific memory CD4+ T cells. Overall, memory CD8^+^ T cell frequencies and response rates were similar between mRNA-1273, BNT162b2, and Ad26.COV2.S immunizations. Low but detectable memory CD8^+^ T cells were observed in some individuals (10-50%) after NVX-CoV2373 immunization. Despite these differences, all vaccines elicited memory CD8^+^ T cells at frequencies comparable to, or slightly higher than, those observed in SARS-CoV-2 recovered individuals at six months (49).

In conclusion, homologous vaccination regimens exhibit considerable variation in immune responses depending on the vaccine platform, particularly concerning antibody titers, B cell activation, and T cell responses. mRNA vaccines, such as mRNA-1273 and BNT162b2, consistently generate higher and more durable levels of neutralizing antibodies and memory B cells compared to adenovirus-based vaccines like Ad26.COV2.S and ChAdOx1. mRNA vaccines also generate robust GC reactions, which persist for months after vaccination thus contributing to sustained antibody production, broadening of the repertoire, and persistent production of activated MBCs. T cell responses, including Th1 and Tfh, are crucial for sustained immunity, with mRNA vaccines showing the most robust CD4+ T cell responses. Adenoviral and protein-based vaccines, induce long-term T cell immunity, especially in CD8+ T cell populations. While all vaccine platforms effectively generate long-term immune memory, mRNA vaccines seem to provide the most comprehensive humoral and cellular immunity.

Antibody concentration, function, subclass, and possible adverse effects have been investigated in individuals who received multiple doses of the same mRNA vaccine. Although the concentration of neutralizing antibodies is used as a correlate of protection, neutralization is not the only function of antibodies. Most spike-specific antibodies (up to 95%) are non-neutralizing (67, 68), but have a protective role. The constant regions of antibody subclasses have different effector functions (69, 70). Vaccination with one or two doses of an mRNA vaccine triggers a humoral response characterized primarily by antibodies of IgG1 and IgG3 subclasses (71). These subclasses are involved in antibody-dependent cytotoxicity, phagocytosis, and complement activation, thus exerting protective functions. It has been shown that after repeated doses, IgG4 antibodies are also produced. Spike-specific IgG4 increased within 6 months following the second dose, with an even greater rise after the third mRNA vaccine dose (72), representing 20% of spike-specific antibodies. IgG4 is a noninflammatory IgG subclass with poor Fc effector functions (73).

IgG4 is the most downstream of the four IgG heavy chain genes and has been detected in the context of repeated antigen exposure (74), indicating that persistent GC responses and repeated stimulation may induce switching to IgG4. This shift toward IgG4 was seen only after mRNA vaccination and not with viral vector and protein-based vaccines (75–77). Although IgG4 has a reduced ability to engage in Fc-mediated effector functions, the neutralization capacity of sera containing IgG4 was not reduced (22). Due to the strong selective pressure exerted by vaccine-induced neutralizing antibodies, the emerging VOCs continuously mutate the RBD region. In this context, spike-specific antibody-mediated antiviral defense may shift to Fc-mediated functions, and a considerable increase of IgG4 may reduce the protective potency of serum antibodies.

## 6 Immunity after heterologous vaccination

Heterologous vaccination was initially considered a strategy to stop the pandemic, given the vaccine shortage at that time, and based on the hypothesis that two different vaccine types may translate into augmented immune responses. A wide variety of heterologous vaccination schemes were subsequently investigated. Initial studies showed that also various heterologous regimens generally gave rise to robust immune responses (78).

Heterologous vaccination strategies, which combine a prime dose from one vaccine platform with a booster from another, have shown conflicting results without a clear benefit in the immune responses compared to homologous vaccination. This approach had initially been speculated to potentially address limitations inherent to individual vaccine platforms. The concept that incorporating vaccines that enhance cellular responses (e.g., viral vector vaccines) with other those that primarily stimulate humoral immunity (e.g., mRNA vaccines and protein-based vaccines), heterologous prime-boost regimens may potentially broaden immunity against SARS-CoV-2 (79).

Studies have consistently shown that individuals primed with ChAdOx1 and boosted with an mRNA vaccine (BNT162b2 or mRNA-1273) achieve much higher neutralizing antibody levels compared to those receiving two doses of ChAdOx1, with antibody titers often comparable to or exceeding those seen in two-dose mRNA regimens (50). Schmidt et al. demonstrated that heterologous vaccination, ChAdOx1 followed with either BNT162b2 or mRNA-1273, induces significantly higher IgG antibody titers against the SARS-CoV-2 spike protein and RBD than homologous ChAdOx1–ChAdOx1 vaccination (80). Participants who received the ChAdOx1–mRNA regimen also exhibited a greater number of circulating spike-specific CD4+ and CD8+ T cells, including cytokine-producing T cells, compared to those in the homologous group (81). Notably, this heterologous strategy compared to homologous strategies (BNT162b2 mRNA-BNT162b2 mRNA or ChAdOx1-ChAdOx1), produced higher CD8+ T cell responses, which are essential for robust antiviral immunity and may provide more durable protection (82). MBC responses were similarly enhanced in ChAdOx1-mRNA recipients, with a greater frequency of spike- and RBD-specific MBCs, especially of the IgG+ isotype and with a higher proportion of activated MBCs (82).

Research has indicated that heterologous vaccination with Ad26.COV2.S followed by an mRNA boost significantly enhanced both the quantity and diversity of spike-specific humoral and cellular immunity which is not focused only on the S1 region of the spike protein (50).

Regarding a heterologous approach using protein vaccine, the randomized multicenter COV-BOOST study was aimed to compare the anti-spike IgG level, viral neutralization and cellular responses after a third booster dose (with 7 different vaccine types) in subjects that received a primary two-dose immunization cycle with either ChAdOx1 or BNT162b2 (83). In the ChAdOx1 primed group, the protein-based NVX-CoV2373 heterologous booster increased anti-spike IgG levels, but not in the BNT162b2 primed group. Similarly, in the T-cell response assay against wild-type and SARS-CoV-2 virus VOC alpha (B.1.1.7), beta (B.1.351), and delta the NVX-CoV2373, heterologous booster improved the result in the group of subjects who received ChAdOx1 priming but not in the BNT162b2 primed ones.

A similar monocentric randomized trial compared heterologous and homologous immune response persistence in ChAdOx1 and BNT162b2 primed cohorts (84). Heterologous ChAdOx1-primed schedules produced and maintained the largest T-cell responses until day 196 post vaccination. Immunization with BNT162b2/NVX-CoV2373 generated a qualitatively different antibody response to BNT162b2/BNT162b2, with the total IgG significantly lower than BNT162b2/BNT162b2 during all follow-up time points, but similar levels of neutralizing antibodies. Heterologous ChAdOx1-primed schedules remain more immunogenic over time in comparison to ChAdOx1/ChAdOx1. BNT162b2-primed schedules with a second dose of either mRNA vaccine also remained more immunogenic over time in comparison to BNT162b/NVX-CoV2373.

In another study it has been shown that NVX-CoV2373 modestly increased humoral immunity to Omicron sub-lineages BA.1, BA.4/BA.5, and BA.2.75 in those primed with an mRNA vaccine (85). Furthermore, in the Ad26.COV2.S and mRNA primed groups Th1 cytokine expression from CD4 + T cells was found at baseline with a modest response to a booster dose of NVX-CoV2373. Only the Ad26.COV2.S-primed participants (11/20) had a high proportion of spike-specific Th1 cytokine expression from CD8+ T cells at baseline with little to no boosting effect noted in any primed group.

The randomized trial carried out by Sheng et al. analyzed cellular response after a fourth dose with NVX-CoV2373 or mRNA-based vaccine in 160 healthy volunteers already vaccinated with three mRNA doses (86). There was no significant increase in the frequency of spike protein-specific memory B (CD19+CD20+) cells in either group after booster vaccination compared to that at baseline. The frequencies of TNF-α- or IFN-γ-secreting CD4+ and CD8+ T cells in the NVX-CoV2373 group were not significantly increased, whereas there was a borderline significant increase in the frequencies of TNF-α- or IFN-γ-secreting CD4+ and CD8+ T cells in the mRNA vaccination group.

It possible to speculate that the combination of two distinct vaccine platforms, each targeting different immune pathways, may be more effective in promoting long-lasting B cell responses and potent T cell responses. However, at present, it is not possible to confirm that a specific heterologous approach is beneficial over others due to high heterogeneity in the study conducted; a meta-analysis by Au et al. aimed to evaluate the effectiveness of heterologous and homologous covid-19 vaccine regimen, identified 63 studies with 25 combinations of COVID-19 vaccine regimens (87).

Studies from the first years of the pandemic has shown that heterologous schedule combining mRNA and adenoviral vaccines may be more immunogenic. Heterologous vaccination strategies, combining sequentially viral vector and mRNA may deliver a more balanced and robust humoral and cellular immune response compared to homologous regimens (88, 89). Data from combining mRNA and protein-based vaccines remain limited.

Looking at a higher level of analysis, a meta-analysis by Asante et al. evaluated the impact of homologous versus heterologous booster regime on all-cause mortality, laboratory-confirmed symptomatic and severe COVID-19, and serious and non-serious adverse events. The pooled analysis of the 29 RCTs showed no benefit of heterologous over homologous booster regime in the above-mentioned endpoint with a trend over more non-serious adverse events in the heterologous booster regimens (79).

## 7 Hybrid immunity

Hybrid immunity, which arises from SARS-CoV-2 infection preceded or followed by vaccination produces markedly stronger immune responses than either vaccination or infection alone (90). Investigators have observed that the protection against re-infection was higher among individuals, who recovered from previous infection and had also received a dose of vaccine before or post-infection, indicating the potency of hybrid immunity (91).

Studies show that individuals with prior infection who received vaccines, such as mRNA or adenovirus-based vaccine, developed significantly higher levels of neutralizing antibodies that are more potent and long-lasting than those generated by vaccination alone (92–94). Moreover, individuals with hybrid immunity also have higher nasal levels of IgG and IgA (55, 95), which contribute to extended protection lasting up to 10 months post-vaccination (96). A study by Bowman et al. used a systems serology approach to explore the qualitative differences in antibody responses associated with hybrid immunity (97). It was found that people with hybrid immunity exhibited superior antibody responses linked to enhanced Fc-receptor binding with a higher capacity for viral clearance. This type of antibody targets the S2 epitopes of the spike protein which is highly conserved across the viral variants.

In terms of B cell responses, hybrid immunity generates a higher frequency of MBCs. RBD-specific MBCs generated in individuals with hybrid immunity have more somatic mutations and show higher affinity maturation than those generated by vaccination only (33, 54, 98). These MBCs persist longer and have greater cross-reactivity against VOCs than those in vaccinated or infected-only individuals (33, 99, 100).

CD4+ and CD8+ T-cell specific immunity is induced by vaccination only and hybrid immunity (90, 101). However, in individuals with previous infection, vaccination reactivates CD4+ T cells with a distinct cytokine profile, producing IFN-γ and IL-10, different from those detected in subjects without previous infection. Thus, hybrid immunity generates spike-specific memory CD4^+^ T cells with imprinted features of SARS-CoV-2 infection. The authors hypothesized that, during infection, IFN-γ drives antiviral responses, whereas IL-10, an anti-inflammatory cytokine, may inhibit the acute inflammation typical of severe that COVID-19.

The protective advantage of hybrid immunity likely arises from a combination of higher numbers of SARS-CoV-2-specific MBCs, elevated neutralizing antibody titers, and an infection-induced cytokine profile of IFN-γ and IL-10 in CD4+ T cells. These immune features were less prominently induced in individuals who received only vaccination, even after a third antigen exposure (90).

## 8 Variants of concern

The emergence of SARS-CoV-2 variants of concern has significantly impacted vaccination strategies and immunity development. Since the original virus emerged in late 2019, several variants have arisen (102), such as Alpha, Beta, Gamma, Delta, and Omicron, each with mutations that affect transmissibility, virulence, and immune escape (103) (**Table 2**). These variants partially evade immunity induced by previous infections or vaccines, prompting the need for updated vaccines and booster doses to enhance protection (104). For instance, while early vaccines were highly effective against the original strain, the emergence of the Delta and especially Omicron variant, with numerous spike mutations led to reduced vaccine efficacy (105). The evolution of COVID-19-specific immunity has been strongly influenced by vaccination and re-infection. The global COVID-19 vaccination campaign was the largest public health campaign in history, with over 2 billion people fully vaccinated within the first 8 months (106).

**Table 2:** Main variants since SARS-CoV-2 outbreak, first date and country of identification and Spike mutation of interest.

Variant Name	Detection Date	Detection Location	Spike Mutations of Interest
Alpha (B.1.1.7)	September 2020	United Kingdom	N501Y, D614G, P681H
Beta (B.1.351)	September 2020	South Africa	K417N, E484K, N501Y, D614G, A701V
Gamma (P.1)	December 2020	Brazil	K417T, E484K, N501Y, D614G, H655Y
Delta (B.1.617.2)	December 2020	India	L452R, T478K, D614G, P681R
Omicron (B.1.1.529)	November 2021	South Africa	G339D, S371L, S373P, S375F, K417N, N440K, G446S, S477N, T478K, E484A, Q493R, G496S, Q498R, N501Y, Y505H
Omicron BA.4	January 2022	South Africa	L452R, F486V, R493Q
Omicron BA.5	February 2022	South Africa	L452R, F486V, R493Q
Omicron BQ.1 (Cerberus)	July 2022	Nigeria	K444T and N460K (168)
Omicron XBB.1.5	October 2022	USA	Q183E, F486P, F490S (169)
Omicron BA.2.86	July 2023	Israel Denmark	The number of spike amino acid mutations in the BA.2.86 variant relative to BA.2 and XBB.1.5 is comparable to the number of mutations in the first Omicron strains relative to the SARS-CoV-2 index strain (170)
Omicron BA.2.87.1	September 2023	USA	G75D, S98F, V126A, W152L, R190S, K417T, K444N, V445G, L452M, N481K, V642G, K679R, S691P, T791I, Y796H, D936G *(mutations based on a limited number of genomes)*
Omicron JN.1	September 2023	Luxembourg	L455S (171)
Omicron KP.2 (JN.1.11.1.2)	April, 2024	India	R346T, F456L (172, 173), V1104L (174)

The immune response to SARS-CoV-2 VOCs varies depending on both the vaccine platform and prior infection status. Hybrid immunity leads to a broader antibody repertoire, providing enhanced neutralization of VOCs (107, 108). Early studies revealed that individuals vaccinated with mRNA vaccines displayed neutralizing antibody and MBC responses like those who had recovered from infection (98). However, the activity of these antibodies was reduced against variants harboring mutations like K417N, E484K, and N501Y, underscoring the need for vaccine updates to combat emerging VOCs. Neutralizing antibodies generated by mRNA vaccines (mRNA-1273 and BNT162b2) show a 20- to 40-fold decrease in potency against Omicron compared to the original strain. However, individuals with hybrid immunity or those who received heterologous vaccination (e.g., ChAdOx1 primed with an mRNA boost) demonstrate improved neutralization against Alpha, Beta, Gamma, and Omicron variants (109–111).

Kotaki et al. found that individuals who received two doses of BNT162b2 produced neutralizing antibodies with limited activity against Beta and Omicron (112). Over time, however, the B cell repertoire expanded to include antibodies that recognized these variants (113). Moreover, a third dose or breakthrough infection further broadened neutralizing antibody activity. For instance, boosting with Omicron or Beta/Delta-targeted mRNA vaccines led to robust GC B cell responses capable of targeting additional variants. A third vaccine dose has been associated with enhanced potency and breadth of neutralizing antibodies, comparable to what is seen in hybrid immunity (114). Continued evolution and expansion of GCs, as well as the emergence of new clones effective against variants like Omicron, were observed after booster doses (115).

T-cell responses, particularly those of CD8+ T cells, remain robust against VOCs (116). Studies show that T cells, especially those induced by the Ad26.COV2.S vaccine, recognize epitopes in all variants, including Delta and Omicron (116). The resilience of T-cell defenses is explained by the observation that T-cell epitopes are less susceptible to mutations than those targeted by neutralizing antibodies. In addition, high affinity is not required for T-cell recognition and function (116). As a result, T cell-mediated immunity plays a key role in protecting against severe disease caused by VOCs, even when antibody efficacy diminishes.

A recent study reported that spike-specific CD4+ and CD8+ T cells from individuals with previous infection or vaccination displayed strong immune recognition of the highly mutated BA.2.86 variant (117). Additionally, different vaccine platforms (mRNA, adenovirus, protein-based) were found to induce memory T cells capable of recognizing a broad range of variants from Alpha to Omicron. In the same individuals, B-cell recognition of the Omicron RBD was reduced by 40%, highlighting the critical role of memory T cells in defending against future infections.

Thus, the emergence of SARS-CoV-2 VOCs has posed significant challenges to vaccination strategies and the development of immunity. The mutations present among VOCs have led to partial evasion of immune responses, necessitating updates to vaccine formulation and additional booster doses to maintain protection. Whereas neutralizing antibodies generated by early vaccines show reduced potency against highly mutated variants, the expansion of the B cell repertoire and booster-induced GC responses (because of infection or additional vaccine dose) adapt antibodies to new variants. Additionally, T-cell-mediated immunity remains robust and crucial for protection against severe outcomes, as T-cell recognition of viral epitopes is less affected by mutations compared to neutralizing antibodies. This resilience, coupled with continued vaccine updates and global immunization efforts, has been vital in managing the impact of evolving variants and safeguarding public health.

## 9 Adolescents/children

SARS-CoV-2 mRNA vaccines were authorized for adolescents (ages 12-15) in May 2021, followed by the approval for children aged 5 to 11 in October 2021, and for those aged 6 to 59 months in June 2022 (118). For children aged 5-11 years, the vaccine dosage is 10 µg for BNT162b2 and 50 µg for mRNA-1273. In younger children (6 months to 4 years), the dosages are lower: 3 µg for BNT162b2 and 25 µg for mRNA-1273 (119, 120).

Real-world data showed that the vaccine's effectiveness against infection was approximately 71% in adolescents, with higher protection rates against COVID-19, reaching 92.1% for mild cases and 97.9% for severe cases (121). However, in children aged 5-11 years, vaccine effectiveness against infection was lower at 44% (122), likely due to the emergence of the Omicron variant at the time of vaccination. Nevertheless, mRNA vaccination reduced the risk of Omicron-related hospitalization by two-thirds in this age group (123).

Bartsch and colleagues evaluated the immune response in children aged 5-11 years who received the BNT162b2 vaccine, in comparison with adolescents (12-15 years) and adults (124). Although the overall antibody levels were lower in children, they demonstrated a stronger neutralization capacity and enhanced opsonophagocytic activity than antibodies of adolescents and adults. Zhong et al. reported that children developed robust antibody, memory B and T-cell responses after two doses of BNT162b2 (125). Children mounted higher antibody and T-cell responses than adults measured 6 months after vaccination. However, a booster dose primarily boosted antibody levels without significantly affecting MBC or T-cell responses.

Our research on the BNT162b2 primary vaccination series in children aged 5-11 years, including both infection-naive and previously infected individuals, showed that children with hybrid immunity (prior infection plus vaccination) had higher levels of spike-specific antibodies, T-cells, and MBCs (126). These children were also capable of producing neutralizing antibodies and T-cell responses against the Omicron variant, despite not having been exposed to it before.

Data on toddlers aged 6 months to 5 years are limited. Nziza et al. investigated antibody levels and their neutralization capacity one month after the second dose of mRNA-1273 in a cohort of 18 young children (127). The total anti-Spike and anti-RBD IgG levels, as well as IgG subclasses, in young children were similar to those in adults, and their neutralization capacity was comparable to that of vaccinated adults. This study also assessed antibody effector functions, such as antibody-dependent cellular phagocytosis by monocytes (ADCP), antibody-dependent neutrophil phagocytosis (ADNP), and antibody-dependent complement deposition or activation (ADCD). Anti-RBD antibodies of young children exhibited stronger ADCP and ADNP activities compared to adults. Additionally, the study showed that these antibodies were effective against VOCs such as Alpha, Beta, Gamma, and Delta, although Omicron-specific immunity was weaker, consistent with findings by Bartsch et al. (128).

Viral vector vaccines (Vaxzevria and Jcovden) were approved only for adults (129, 130) and therefore no data have been generated in adolescents. Similarly, Bimervax is not recommended for people below 16 years of age. Currently, there is no information available on the use of Bimervax in children younger than 16 years of age (131). The other protein-based vaccine, Nuvaxovid and its adapted vaccines are not currently authorized for use in children below 12 years of age (132). A main study including over 2,200 children aged 12 to 17 years is being carried out in accordance with the pediatric investigation plan (PIP) for the vaccine, which was agreed by EMA's Pediatric Committee (PDCO).This trial showed that the immune response to Nuvaxovid in adolescents, which was measured as the level of antibodies against SARS-CoV-2, was comparable to the response in young adults aged 18 to 25 years (133). In conclusion, mRNA vaccines have shown varying levels of effectiveness in children across different age groups, with the strongest protection observed in adolescents. Although vaccine effectiveness against infection is lower in younger children, especially with the emergence of the Omicron variant, vaccination still significantly reduces the risk of severe disease and hospitalization. Studies indicate that children mount robust immune responses, including strong antibody effector function and T-cell activity, which may be further enhanced in individuals with hybrid immunity. However, there is still limited data on toddlers, highlighting the need for further research to investigate vaccine-induced immunity in the youngest age groups and to optimize vaccination strategies.

## 10 Aged and immunocompromised patients

Although the morbidity and mortality of COVID-19 have been drastically reduced by vaccination, immunocompromised patients remain a high-risk group (134). It has been calculated that 3-6% of the population in high-income countries has an impaired immune system because of immunosuppressive treatments, cancer, bone marrow transplantation, or primary immunodeficiency (135, 136). A much larger fraction of persons have an increased risk of dying of COVID-19, simply because of old age (137). The combined effects of immune aging and comorbidities render the elderly susceptible to severe COVID-19 and, compared to the younger population, people over 75 have a 9-fold increased risk of dying from COVID-19 (137).

Ageing is associated with impaired antigen presentation, T cell priming and germinal center function (138). Thus, because of the impaired adaptive immune responses, which can impact the vaccine efficacy, the elderly are predisposed to a worsen disease outcome (139). Moreover, the continued SARS-CoV-2 circulation has led to the development of more infectious and transmissible variants.

It has been found that older individuals exhibited a less robust adaptive immune response compared to younger individuals following vaccination with the BNT162b2 mRNA vaccine. The neutralization capacity was inversely correlated with age and worsen after the age of 80 years (140). Ageing was also associated with a lower concentration of antibodies against RBD and a reduced frequency of SARS-CoV-2 specific T cells able to produce two different cytokines (141, 142). This underscores the importance of tailored vaccination strategies and possibly booster doses to enhance immunity in the elderly.

Patients with immune-related conditions, such as inborn errors of immunity, cancer, and those undergoing transplantation, face significant risks when it comes to COVID-19 due to compromised immune systems that may not respond effectively to vaccination. For individuals with inborn errors of immunity, these genetic conditions result in fundamental defects in immune cell development or function, leading to a diminished ability to generate a robust immune response after vaccination (143). This population's reduced capacity for mounting a protective immune response against COVID-19 underscores the need for specialized vaccination strategies (143).

Cancer patients, particularly those undergoing treatments such as chemotherapy or immunotherapy, often experience immune suppression due to both the malignancy itself and the therapeutic regimens used to control it. Chemotherapy depletes immune cells, while immunotherapy, though aimed at enhancing immune responses against cancer, can paradoxically induce immune dysfunction. These factors lead to a reduced ability to generate vaccine-induced immunity, making cancer patients more vulnerable to severe outcomes if infected with SARS-CoV-2. Immunological monitoring and adapting vaccination schedules, including additional doses or boosters, have been suggested as strategies to enhance vaccine effectiveness in these patients (144, 145).

Transplant recipients, such as those undergoing organ or allogeneic stem cell transplantation, represent another group at high risk. Immunosuppressive therapies used to prevent organ rejection or graft-versus-host disease significantly dampen the immune system's ability to respond to vaccines. The weakened immune response persists for a prolonged period post-transplant, posing challenges to achieving adequate vaccine protection. Studies indicate that alternative approaches, such as pre-emptive immunization before transplantation or repeated booster doses, may help improve immune responses and provide better protection in this population (146, 147).

Further complicating these challenges, immune-related conditions like autoimmune diseases or multiple sclerosis also necessitate the use of immunosuppressive or immunomodulatory therapies, which can negatively impact the vaccine response. Agents used to control autoimmune inflammation, such as corticosteroids or biologics, can reduce vaccine immunogenicity, necessitating personalized vaccination strategies to achieve optimal protection (148, 149).

Assessing vaccine efficacy in immunocompromised patients is crucial, as these individuals often exhibit a diminished ability to generate protective immune responses due to underlying conditions or immunosuppressive treatments. Accurate readouts of vaccine efficacy, such as measurements of antibody titers, T-cell and B-cell responses, and clinical outcomes like infection rates and disease severity, are essential to evaluate how well vaccines protect this vulnerable population. For immunocompromised patients, standard vaccine response metrics used in the general population may not provide an accurate picture of protection, making tailored approaches to efficacy evaluation necessary. These readouts guide clinical decision-making, including the need for booster doses or alternative vaccination strategies, helping to ensure that immunocompromised individuals receive adequate protection against COVID-19 (145).

Research supports the idea that optimizing vaccination strategies tailored to these at-risk groups, such as timing vaccines to coincide with lower levels of immunosuppression, utilizing novel vaccine platforms, or incorporating additional booster doses, can help enhance immune responses. Careful consideration of each patient’s unique immunological profile and treatment regimen is essential for maximizing the protective benefits of COVID-19 vaccination (150–152). In October 2024, ACIP recommended that all persons aged ≥65 years and persons aged 6 months–64 years with moderate or severe immunocompromise receive a recall dose of the 2024–2025 COVID-19 vaccine 6 months after their last dose (153).

Interestingly, among immunocompromised patients a recent meta-analysis by Pardo et al. summarized the outcome of different heterologous and homologous vaccinations assessing IgG and neutralizing antibody production as well as vaccine effectiveness (154). In this setting, considering an intrinsic variability in study design and analytical methods, heterologous COVID-19 vaccines showed comparable rates of antibody response and effectiveness compared to homologous strategies.

## 11 Conclusions

As SARS-CoV-2 continues to evolve, it is essential to explore optimal vaccination strategies that maintain robust and durable immune protection. mRNA and protein-based vaccines have been demonstrated to generate high levels of neutralizing antibodies. Compared to other vaccines, mRNA vaccines induced strong memory B and Tfh cell responses. However, adenovirus vector vaccines offer advantages in generating durable T cell responses, which may be critical for long-term protection against VOC. Heterologous vaccination and hybrid immunity may provide further opportunities to enhance immune responses, offering broader protection and greater durability of immunity. These strategies may be especially important to maintain effective immune responses in the face of rapidly evolving viral variants. It is crucial to underline that at present, due to many variables (viral strain, vaccination schedule, vaccine availability, serological status) it is difficult to identify a heterologous schedule that undoubtedly will provide long-lasting immunological memory. However, in the current scenario, with almost the entire global population having been exposed to SARS-CoV-2 or being effectively vaccinated, severe COVID-19 outcomes, independently from the type of immunization are effectively prevented by staying up to date with recommended doses of strain adapted vaccines. A validated and internationally accepted correlate of protection has not been identified so far. Although neutralizing antibody levels seems to better correlate with vaccine efficacy, other immune responses may also play a substantial role in protection against progression from symptomatic to severe SARS-CoV-2 infection (155). Continued research into the development of next-generation vaccines and booster strategies will be key to achieving sustained immunity and controlling the severe outcomes of SARS-CoV-2 infection.

## 12 Future perspectives on vaccination strategies

Parenteral vaccines have successfully reduced severe outcomes of COVID-19, but fail to induce sterilizing immunity (55, 156). Systemic vaccines are unable to establish long-lasting tissue-resident immune cells in the respiratory mucosa (157–162), suggesting that local immunization strategies may be more successful in fostering mucosal immunity.

Future perspectives on mucosal vaccines are promising, especially in the context of respiratory infections like COVID-19. Mucosal vaccines, administered through routes such as nasal sprays, aim to stimulate local immune responses at the site of pathogen entry, offering a first line of defense. This approach has the potential to generate robust mucosal immunity, characterized by the production of secretory IgA antibodies and tissue-resident memory T cells, which can rapidly respond to reinfection. Unlike traditional intramuscular vaccines that primarily elicit systemic immunity, mucosal vaccines may provide superior protection against infection and transmission by targeting the respiratory tract directly (163). Additionally, they hold promise for overcoming current vaccine limitations in inducing durable immunity and might be especially beneficial for immunocompromised patients who struggle to mount effective responses to conventional vaccines. Ongoing research is focused on optimizing delivery methods and formulations to enhance the efficacy and safety of mucosal vaccines for widespread use (163). Collectively, mucosal vaccine strategies may provide more robust sterilizing immunity to block SARS-CoV-2 transmission and diminish the capacity for SARS-CoV-2 immune evasion mutants to emerge (164). However, given the ability of intramuscular mRNA vaccination to boost pre-existing mucosal immunity and the high prevalence of SARS-CoV-2 breakthrough infection, mucosal vaccines may exhibit minimal advantage over current mRNA vaccine platforms in the context of a population with high rates of prior SARS-CoV-2 infection (164). More clinical data on mucosal vaccine efficacy is needed, including comparisons to approved mRNA vaccines in previously infected subjects and in those with no prior infection. Besides mucosal vaccine, other strategies are under evaluation aimed at achieving improved immunity, namely Self-Amplifying mRNA, pan SARS-COV vaccines, and combined vaccines (165). Furthermore, next-generation vaccines could involve tweaks to these designs or changes to delivery mechanisms that might improve performance.

As COVID-19 is no longer seen as the all-encompassing emergency it once was, developers and regulators will move more slowly compared with the breakneck pace of the first-generation vaccines. There is no need to rush these through because what it will be crucial is to generate vaccines that will be able to elicit long and durable protection against COVID-19.

## References

[1] Chan JF Yuan S Kok KH To KK Chu H Yang J. A familial cluster of pneumonia associated with the 2019 novel coronavirus indicating person-to-person transmission: a study of a family cluster. Lancet. (2020) 395:514–23. doi: 10.1016/S0140-6736(20)30154-9 PMC7159286 31986261

[2] Huang C Wang Y Li X Ren L Zhao J Hu Y. Clinical features of patients infected with 2019 novel coronavirus in Wuhan, China. Lancet. (2020) 395:497–506. doi: 10.1016/S0140-6736(20)30183-5 31986264 PMC7159299

[3] Zhu N Zhang D Wang W Li X Yang B Song J. A novel coronavirus from patients with pneumonia in China, 2019. N Engl J Med. (2020) 382:727–33. doi: 10.1056/NEJMoa2001017 PMC7092803 31978945

[4] WHO. Available online at: https://www.who.int/europe/emergencies/situations/covid-19:~:text=Cases%20of%20novel%20coronavirus%20(nCoV,pandemic%20on%2011%20March%202020 (Accessed July 7 2024).

[5] WHO. Available online at: https://www.who.int/news/item/05-05-2023-statement-on-the-fifteenth-meeting-of-the-international-health-regulations-(2005)-emergency-committee-regarding-the-coronavirus-disease-(covid-19)-pandemic?adgroupsurvey=%7Badgroupsurvey%7D (Accessed October 30, 2024).

[6] CEPI. Available online at: https://cepi.net/cepi-20-and-100-days-mission (Accessed October 30, 2024).

[7] Baden LR El Sahly HM Essink B Kotloff K Frey S Novak R. Efficacy and safety of the mRNA-1273 SARS-coV-2 vaccine. N Engl J Med. (2021) 384:403–16. doi: 10.1056/NEJMoa2035389 PMC7787219 33378609

[8] Polack FP Thomas SJ Kitchin N Absalon J Gurtman A Lockhart S. Safety and efficacy of the BNT162b2 mRNA covid-19 vaccine. N Engl J Med. (2020) 383:2603–15. doi: 10.1056/NEJMoa2034577 PMC7745181 33301246

[9] Sadoff J Gray G Vandebosch A Cardenas V Shukarev G Grinsztejn B. Safety and efficacy of single-dose ad26.COV2.S vaccine against covid-19. N Engl J Med. (2021) 384:2187–201. doi: 10.1056/NEJMoa2101544 PMC8220996 33882225

[10] Voysey M Clemens SAC Madhi SA Weckx LY Folegatti PM Aley PK. Safety and efficacy of the ChAdOx1 nCoV-19 vaccine (AZD1222) against SARS-CoV-2: an interim analysis of four randomised controlled trials in Brazil, South Africa, and the UK. Lancet. (2021) 397:99–111. doi: 10.1016/S0140-6736(20)32661-1 33306989 PMC7723445

[11] WHO. Available online at: https://www.who.int/europe/news/item/16-01-2024-covid-19-vaccinations-have-saved-more-than-1.4-million-lives-in-the-who-european-region–a-new-study-finds:~:text=Mortality%20in%20countries%20reduced%20by,December%202021%20to%20April%202023 (Accessed October 30, 2024).

[12] Larkin H. Hybrid immunity more protective than prior SARS-coV-2 infection alone. JAMA. (2023) 329:531. doi: 10.1001/jama.2023.0743 36723971

[13] Hattab D Amer MFA Al-Alami ZM Bakhtiar A. SARS-CoV-2 journey: from alpha variant to omicron and its sub-variants. Infection. (2024) 52:767–86. doi: 10.1007/s15010-024-02223-y PMC11143066 38554253

[14] Yang H Rao Z. Structural biology of SARS-CoV-2 and implications for therapeutic development. Nat Rev Microbiol. (2021) 19:685–700. doi: 10.1038/s41579-021-00630-8 34535791 PMC8447893

[15] Lei S Chen X Wu J Duan X Men K. Small molecules in the treatment of COVID-19. Signal Transduct Target Ther. (2022) 7:387. doi: 10.1038/s41392-022-01249-8 36464706 PMC9719906

[16] Malone B Urakova N Snijder EJ Campbell EA. Structures and functions of coronavirus replication–transcription complexes and their relevance for SARS-CoV-2 drug design. Nat Rev Mol Cell Biol. (2022) 23:21–39. doi: 10.1038/s41580-021-00432-z 34824452 PMC8613731

[17] V’kovski P Kratzel A Steiner S Stalder H Thiel V. Coronavirus biology and replication: implications for SARS-CoV-2. Nat Rev Microbiol. (2021) 19:155–70. doi: 10.1038/s41579-020-00468-6 PMC7592455 33116300

[18] Sergio MC Ricciardi S Guarino AM Giaquinto L De Matteis MA. Membrane remodeling and trafficking piloted by SARS-CoV-2. Trends Cell Biol. (2024) 34:785–800. doi: 10.1016/j.tcb.2023.12.006 38262893

[19] Marano V Vlachová Š Tiano SML Cortese M. A portrait of the infected cell: how SARS-CoV-2 infection reshapes cellular processes and pathways. NPJ Virus. (2024) 2:66. doi: 10.1038/s44298-024-00076-8 PMC11721117 40295886

[20] Del Valle DM Kim-Schulze S Huang HH Beckmann ND Nirenberg S Wang B. An inflammatory cytokine signature predicts COVID-19 severity and survival. Nat Med. (2020) 26:1636–43. doi: 10.1038/s41591-020-1051-9 PMC7869028 32839624

[21] Gray PE Bartlett AW Tangye SG. Severe COVID-19 represents an undiagnosed primary immunodeficiency in a high proportion of infected individuals. Clin Transl Immunol. (2022) 11:e1365. doi: 10.1002/cti2.v11.4 PMC9013505 35444807

[22] Lapuente D Winkler TH Tenbusch M. B-cell and antibody responses to SARS-CoV-2: infection, vaccination, and hybrid immunity. Cell Mol Immunol. (2024) 21:144–58. doi: 10.1038/s41423-023-01095-w PMC10805925 37945737

[23] Primorac D Vrdoljak K Brlek P Pavelic E Molnar V Matisic V. Adaptive immune responses and immunity to SARS-coV-2. Front Immunol. (2022) 13:848582. doi: 10.3389/fimmu.2022.848582 35603211 PMC9114812

[24] Victora GD Nussenzweig MC. Germinal centers. Annu Rev Immunol. (2022) 40:413–42. doi: 10.1146/annurev-immunol-120419-022408 35113731

[25] Elsner RA Shlomchik MJ. Germinal center and extrafollicular B cell responses in vaccination, immunity, and autoimmunity. Immunity. (2020) 53:1136–50. doi: 10.1016/j.immuni.2020.11.006 PMC7748291 33326765

[26] Kaneko N Kuo HH Boucau J Farmer JR Allard-Chamard H Mahajan VS. Loss of bcl-6-expressing T follicular helper cells and germinal centers in COVID-19. Cell. (2020) 183:143–57 e13. doi: 10.1016/j.cell.2020.08.025 32877699 PMC7437499

[27] Woodruff MC Ramonell RP Nguyen DC. Extrafollicular B cell responses correlate with neutralizing antibodies and morbidity in COVID-19. Nat Immunol. (2020) 21:1506–16. doi: 10.1038/s41590-020-00814-z PMC7739702 33028979

[28] Dan JM Mateus J Kato Y Hastie KM Yu ED Faliti CE. Immunological memory to SARS-CoV-2 assessed for up to 8 months after infection. Science. (2021) 371. doi: 10.1126/science.abf4063 PMC7919858 33408181

[29] Gaebler C Wang Z Lorenzi JCC Muecksch F Finkin S Tokuyama M. Evolution of antibody immunity to SARS-CoV-2. Nature. (2021) 591:639–44. doi: 10.1038/s41586-021-03207-w PMC8221082 33461210

[30] Muecksch F Weisblum Y Barnes CO. Affinity maturation of SARS-CoV-2 neutralizing antibodies confers potency, breadth, and resilience to viral escape mutations. Immunity. (2021) 54:1853–68 e7. doi: 10.1016/j.immuni.2021.07.008 34331873 PMC8323339

[31] Sakharkar M Rappazzo CG Wieland-Alter WF Hsieh CL Wrapp D Esterman ES. Prolonged evolution of the human B cell response to SARS-CoV-2 infection. Sci Immunol. (2021) 6. doi: 10.1126/sciimmunol.abg6916 PMC8128290 33622975

[32] Sokal A Chappert P Barba-Spaeth G. Maturation and persistence of the anti-SARS-CoV-2 memory B cell response. Cell. (2021) 184:1201–13 e14. doi: 10.1016/j.cell.2021.01.050 33571429 PMC7994111

[33] Wang Z Muecksch F Schaefer-Babajew D Finkin S Viant C Gaebler C. Naturally enhanced neutralizing breadth against SARS-CoV-2 one year after infection. Nature. (2021) 595:426–31. doi: 10.1038/s41586-021-03696-9 PMC8277577 34126625

[34] Laidlaw BJ Ellebedy AH. The germinal centre B cell response to SARS-CoV-2. Nat Rev Immunol. (2022) 22:7–18. doi: 10.1038/s41577-021-00657-1 34873279 PMC8647067

[35] Rodda LB Netland J Shehata L Pruner KB Morawski PA Thouvenel CD. Functional SARS-coV-2-specific immune memory persists after mild COVID-19. Cell. (2021) 184:169–83.e17. doi: 10.1016/j.cell.2020.11.029 33296701 PMC7682481

[36] Inoue T Kurosaki T. Memory B cells. Nat Rev Immunol. (2024) 24:5–17. doi: 10.1038/s41577-023-00897-3 37400644

[37] Schaefer-Babajew D Wang Z Muecksch F Cho A Loewe M Cipolla M. Antibody feedback regulates immune memory after SARS-CoV-2 mRNA vaccination. Nature. (2023) 613:735–42. doi: 10.1038/s41586-022-05609-w PMC9876794 36473496

[38] Sherina N Piralla A Du L Wan H Kumagai-Braesch M Andrell J. Persistence of SARS-CoV-2-specific B and T cell responses in convalescent COVID-19 patients 6-8 months after the infection. Med. (2021) 2:281–95 e4. doi: 10.1016/j.medj.2021.02.001 33589885 PMC7874960

[39] Zuo J Dowell AC Pearce H Verma K Long HM Begum J. Robust SARS-CoV-2-specific T cell immunity is maintained at 6 months following primary infection. Nat Immunol. (2021) 22:620–6. doi: 10.1038/s41590-021-00902-8 PMC7610739 33674800

[40] Nesamari R Omondi MA Baguma R Höft MA Ngomti A Nkayi AA. Post-pandemic memory T&xa0;cell response to SARS-CoV-2 is durable, broadly targeted, and cross-reactive to the hypermutated BA.2.86 variant. Cell Host Microbe. (2024) 32:162–9.e3. doi: 10.1016/j.chom.2023.12.003 38211583 PMC10901529

[41] Le Bert N Tan AT Kunasegaran K Tham CYL Hafezi M Chia A. SARS-CoV-2-specific T cell immunity in cases of COVID-19 and SARS, and uninfected controls. Nature. (2020) 584:457–62. doi: 10.1038/s41586-020-2550-z 32668444

[42] Sievers BL Cheng MTK Csiba K Meng B Gupta RK. SARS-CoV-2 and innate immunity: the good, the bad, and the "goldilocks. Cell Mol Immunol. (2024) 21:171–83. doi: 10.1038/s41423-023-01104-y PMC10805730 37985854

[43] Kavathas PB Krause PJ Ruddle NH. Adaptive Immunity: Antigen Recognition by T and B Lymphocytes. In: Krause PJ Kavathas PB Ruddle NH, editors. Immunoepidemiology. Springer International Publishing, Cham (2019). p. 55–74.

[44] Stokel-Walker C. What do we know about the adaptive immune response to covid-19? BMJ. (2023) 380:19. 36717121 10.1136/bmj.p19

[45] Hoehn KB Ramanathan P Unterman A Sumida TS Asashima H Hafler DA. Cutting edge: Distinct B cell repertoires characterize patients with mild and severe COVID-19. J Immunol. (2021) 206:2785–90. doi: 10.4049/jimmunol.2100135 PMC8627528 34049971

[46] Hou H Zhang Y Tang G Luo Y Liu W Cheng C. Immunologic memory to SARS-CoV-2 in convalescent COVID-19 patients at 1 year postinfection. J Allergy Clin Immunol. (2021) 148:1481–92 e2. doi: 10.1016/j.jaci.2021.09.008 34536418 PMC8440318

[47] Byrne J Gu L Garcia-Leon A Gaillard CM Saini G Alalwan D. Robust and persistent B-cell responses following SARS-CoV-2 vaccine determine protection from SARS-CoV-2 infection. Front Immunol. (2024) 15:1445653. doi: 10.3389/fimmu.2024.1445653 39355249 PMC11442242

[48] Yang G Cao J Qin J Mei X Deng S Xia Y. Initial COVID-19 severity influenced by SARS-CoV-2-specific T cells imprints T-cell memory and inversely affects reinfection. Signal Transduct Target Ther. (2024) 9:141. doi: 10.1038/s41392-024-01867-4 38811527 PMC11136975

[49] Zhang Z Mateus J Coelho CH Dan JM Moderbacher CR Galvez RI. Humoral and cellular immune memory to four COVID-19 vaccines. Cell. (2022) 185:2434–51 e17. doi: 10.1016/j.cell.2022.05.022 35764089 PMC9135677

[50] Khoo NKH Lim JME Gill US de Alwis R Tan N Toh JZN. Differential immunogenicity of homologous versus heterologous boost in Ad26.COV2.S vaccine recipients. Med. (2022) 3:104–18 e4. doi: 10.1016/j.medj.2021.12.004 35072129 PMC8767655

[51] Balinsky CA Jiang L Jani V Cheng Y Zhang Z Belinskaya T. Antibodies to S2 domain of SARS-CoV-2 spike protein in Moderna mRNA vaccinated subjects sustain antibody-dependent NK cell-mediated cell cytotoxicity against Omicron BA.1. Front Immunol. (2023) 14:1266829. doi: 10.3389/fimmu.2023.1266829 38077368 PMC10702584

[52] Prather AA Dutcher EG Robinson J Lin J Blackburn E Hecht FM. Predictors of long-term neutralizing antibody titers following COVID-19 vaccination by three vaccine types: the BOOST study. Sci Rep. (2023) 13:6505. doi: 10.1038/s41598-023-33320-x 37160978 PMC10170073

[53] Ciabattini A Pastore G Fiorino F Polvere J Lucchesi S Pettini E. Evidence of SARS-coV-2-specific memory B cells six months after vaccination with the BNT162b2 mRNA vaccine. Front Immunol. (2021) 12:740708. doi: 10.3389/fimmu.2021.740708 34650563 PMC8505800

[54] Sokal A Barba-Spaeth G Fernandez I Broketa M Azzaoui I de La Selle A. mRNA vaccination of naive and COVID-19-recovered individuals elicits potent memory B cells that recognize SARS-CoV-2 variants. Immunity. (2021) 54:2893–907 e5. doi: 10.1016/j.immuni.2021.09.011 34614412 PMC8452492

[55] Terreri S Piano Mortari E Vinci MR Russo C Alteri C Albano C. Persistent B cell memory after SARS-CoV-2 vaccination is functional during breakthrough infections. Cell Host Microbe. (2022) 30:400–8 e4. doi: 10.1016/j.chom.2022.01.003 35134333 PMC8820949

[56] Turner JS O'Halloran JA Kalaidina E Kim W Schmitz AJ Zhou JQ. SARS-CoV-2 mRNA vaccines induce persistent human germinal centre responses. Nature. (2021) 596:109–13. doi: 10.1038/s41586-021-03738-2 PMC8935394 34182569

[57] Chen C Wang X Zhang Z. Humoral and cellular immunity against diverse SARS-CoV-2 variants. J Genet Genomics. (2023) 50:934–47. doi: 10.1016/j.jgg.2023.10.003 37865193

[58] Lee Y Tarke A Grifoni A. In-depth characterization of T cell responses with a combined Activation-Induced Marker (AIM) and Intracellular Cytokine Staining (ICS) assay. Oxf Open Immunol. (2024) 5:iqae014. doi: 10.1093/oxfimm/iqae014 39713046 PMC11661976

[59] Bai J Chiba A Murayama G Kuga T Yahagi Y Tabe Y. Early CD4(+) T cell responses induced by the BNT162b2 SARS-CoV-2 mRNA vaccine predict immunological memory. Sci Rep. (2022) 12:20376. doi: 10.1038/s41598-022-24938-4 36437407 PMC9701808

[60] Barbeau DJ Martin JM Carney E Dougherty E Doyle JD Dermody TS. Comparative analysis of human immune responses following SARS-CoV-2 vaccination with BNT162b2, mRNA-1273, or Ad26.COV2.S. NPJ Vaccines. (2022) 7:77. doi: 10.1038/s41541-022-00504-x 35794181 PMC9258461

[61] Nowill AE Caruso M de-Campos-Lima PO. T-cell immunity to SARS-CoV-2: what if the known best is not the optimal course for the long run? Adapting to evolving targets. Front Immunol. (2023) 14:1133225. doi: 10.3389/fimmu.2023.1133225 37388738 PMC10303130

[62] Crotty S. T follicular helper cell biology: A decade of discovery and diseases. Immunity. (2019) 50:1132–48. doi: 10.1016/j.immuni.2019.04.011 PMC6532429 31117010

[63] Lederer K Bettini E Parvathaneni K Painter MM Agarwal D Lundgreen KA. Germinal center responses to SARS-CoV-2 mRNA vaccines in healthy and immunocompromised individuals. Cell. (2022) 185:1008–24 e15. doi: 10.1016/j.cell.2022.01.027 35202565 PMC8808747

[64] Mudd PA Minervina AA Pogorelyy MV Turner JS Kim W Kalaidina E. SARS-CoV-2 mRNA vaccination elicits a robust and persistent T follicular helper cell response in humans. Cell. (2022) 185:603–13 e15. doi: 10.1016/j.cell.2021.12.026 35026152 PMC8695127

[65] Moyo-Gwete T Richardson SI Keeton R Hermanus T Spencer H Manamela NP. Homologous Ad26.COV2.S vaccination results in reduced boosting of humoral responses in hybrid immunity, but elicits antibodies of similar magnitude regardless of prior infection. PloS Pathog. (2023) 19:e1011772. doi: 10.1371/journal.ppat.1011772 37943890 PMC10684107

[66] Cupovic J Ring SS Onder L Colston JM Lutge M Cheng HW. Adenovirus vector vaccination reprograms pulmonary fibroblastic niches to support protective inflating memory CD8(+) T cells. Nat Immunol. (2021) 22:1042–51. doi: 10.1038/s41590-021-00969-3 PMC7611414 34267375

[67] Bartsch YC Tong X Kang J Avendano MJ Serrano EF Garcia-Salum T. Omicron variant Spike-specific antibody binding and Fc activity are preserved in recipients of mRNA or inactivated COVID-19 vaccines. Sci Transl Med. (2022) 14:eabn9243. doi: 10.1126/scitranslmed.abn9243 35289637 PMC8995028

[68] Kaplonek P Fischinger S Cizmeci D Bartsch YC Kang J Burke JS. mRNA-1273 vaccine-induced antibodies maintain Fc effector functions across SARS-CoV-2 variants of concern. Immunity. (2022) 55:355–65 e4. doi: 10.1016/j.immuni.2022.01.001 35090580 PMC8733218

[69] DiLillo DJ Tan GS Palese P Ravetch JV. Broadly neutralizing hemagglutinin stalk-specific antibodies require FcgammaR interactions for protection against influenza virus *in vivo* . Nat Med. (2014) 20:143–51. doi: 10.1038/nm.3443 PMC3966466 24412922

[70] Gunn BM Yu WH Karim MM Brannan JM Herbert AS Wec AZ. A role for fc function in therapeutic monoclonal antibody-mediated protection against ebola virus. Cell Host Microbe. (2018) 24:221–33 e5. doi: 10.1016/j.chom.2018.07.009 30092199 PMC6298217

[71] Kaplonek P Cizmeci D Fischinger S Collier AR Suscovich T Linde C. mRNA-1273 and BNT162b2 COVID-19 vaccines elicit antibodies with differences in Fc-mediated effector functions. Sci Transl Med. (2022) 14:eabm2311. doi: 10.1126/scitranslmed.abm2311 35348368 PMC8995030

[72] Irrgang P Gerling J Kocher K Lapuente D Steininger P Habenicht K. Class switch toward noninflammatory, spike-specific IgG4 antibodies after repeated SARS-CoV-2 mRNA vaccination. Sci Immunol. (2023) 8:eade2798. doi: 10.1126/sciimmunol.ade2798 36548397 PMC9847566

[73] Rispens T Huijbers MG. The unique properties of IgG4 and its roles in health and disease. Nat Rev Immunol. (2023) 23:763–78. doi: 10.1038/s41577-023-00871-z PMC10123589 37095254

[74] Boonpiyathad T Meyer N Moniuszko M Sokolowska M Eljaszewicz A Wirz OF. High-dose bee venom exposure induces similar tolerogenic B-cell responses in allergic patients and healthy beekeepers. Allergy. (2017) 72:407–15. doi: 10.1111/all.12966 27341567

[75] Buhre JS Pongracz T Kunsting I Lixenfeld AS Wang W Nouta J. mRNA vaccines against SARS-CoV-2 induce comparably low long-term IgG Fc galactosylation and sialylation levels but increasing long-term IgG4 responses compared to an adenovirus-based vaccine. Front Immunol. (2022) 13:1020844. doi: 10.3389/fimmu.2022.1020844 36713457 PMC9877300

[76] Kiszel P Sik P Miklos J Kajdacsi E Sinkovits G Cervenak L. Class switch towards spike protein-specific IgG4 antibodies after SARS-CoV-2 mRNA vaccination depends on prior infection history. Sci Rep. (2023) 13:13166. doi: 10.1038/s41598-023-40103-x 37574522 PMC10423719

[77] Kalkeri R Zhu M Cloney-Clark S Plested JS Parekh A Gorinson D. Altered IgG4 antibody response to repeated mRNA versus recombinant protein SARS-CoV-2 vaccines. J Infect. (2024) 88:106119. doi: 10.1016/j.jinf.2024.106119 38360356

[78] Ho TC Chen YA Chan HP Chang CC Chuang KP Lee CH. The effects of heterologous immunization with prime-boost COVID-19 vaccination against SARS-coV-2. Vaccines (Basel). (2021) 9. doi: 10.3390/vaccines9101163 PMC8537265 34696271

[79] Asante MA Michelsen ME Balakumar MM Kumburegama B Sharifan A Thomsen AR. Heterologous versus homologous COVID-19 booster vaccinations for adults: systematic review with meta-analysis and trial sequential analysis of randomised clinical trials. BMC Med. (2024) 22:263. doi: 10.1186/s12916-024-03471-3 38915011 PMC11197367

[80] Schmidt T Klemis V Schub D Schneitler S Reichert MC Wilkens H. Cellular immunity predominates over humoral immunity after homologous and heterologous mRNA and vector-based COVID-19 vaccine regimens in solid organ transplant recipients. Am J Transplant. (2021) 21:3990–4002. doi: 10.1111/ajt.16818 34453872 PMC8652989

[81] Schmidt T Klemis V Schub D Mihm J Hielscher F Marx S. Immunogenicity and reactogenicity of heterologous ChAdOx1 nCoV-19/mRNA vaccination. Nat Med. (2021) 27:1530–5. doi: 10.1038/s41591-021-01464-w PMC8440177 34312554

[82] Pozzetto B Legros V Djebali S Barateau V Guibert N Villard M. Immunogenicity and efficacy of heterologous ChAdOx1-BNT162b2 vaccination. Nature. (2021) 600:701–6. doi: 10.1038/s41586-021-04120-y 34673755

[83] Munro APS Janani L Cornelius V Aley PK Babbage G Baxter D. Safety and immunogenicity of seven COVID-19 vaccines as a third dose (booster) following two doses of ChAdOx1 nCov-19 or BNT162b2 in the UK (COV-BOOST): a blinded, multicentre, randomised, controlled, phase 2 trial. Lancet. (2021) 398:2258–76. doi: 10.1016/S0140-6736(21)02717-3 PMC8639161 34863358

[84] Shaw RH Greenland M Stuart ASV Aley PK Andrews NJ Cameron JC. Persistence of immune response in heterologous COVID vaccination schedules in the Com-COV2 study - A single-blind, randomised trial incorporating mRNA, viral-vector and protein-adjuvant vaccines. J Infect. (2023) 86:574–83. doi: 10.1016/j.jinf.2023.03.027 PMC10076082 37028454

[85] Lyke KE Atmar RL Dominguez Islas C Posavad CM Deming ME Branche AR. Immunogenicity of NVX-CoV2373 heterologous boost against SARS-CoV-2 variants. NPJ Vaccines. (2023) 8:98. doi: 10.1038/s41541-023-00693-z 37433788 PMC10336079

[86] Sheng WH Lin PH Cheng YC Wu YY Hsieh MJ Yang HC. Immunogenicity and safety of heterologous booster with protein-based COVID-19 vaccine (NVX-CoV2373) in healthy adults: A comparative analysis with mRNA vaccines. J Formos Med Assoc. (2024) 123:340–6. doi: 10.1016/j.jfma.2023.10.012 37996322

[87] Au WY Cheung PP. Effectiveness of heterologous and homologous covid-19 vaccine regimens: living systematic review with network meta-analysis. BMJ. (2022) 377:e069989. doi: 10.1136/bmj-2022-069989 35640925 PMC9724446

[88] Culebras E Martinez M Novella C Leon JM Marcos E Delgado-Iribarren A. Cell immunity to SARS-CoV-2 after natural infection and/or different vaccination regimens. Front Cell Infect Microbiol. (2024) 14:1370859. doi: 10.3389/fcimb.2024.1370859 38572317 PMC10987831

[89] Lv J Wu H Xu J Liu J. Immunogenicity and safety of heterologous versus homologous prime-boost schedules with an adenoviral vectored and mRNA COVID-19 vaccine: a systematic review. Infect Dis Poverty. (2022) 11:53. doi: 10.1186/s40249-022-00977-x 35562753 PMC9100319

[90] Rodda LB Morawski PA Pruner KB Fahning ML Howard CA Franko N. Imprinted SARS-CoV-2-specific memory lymphocytes define hybrid immunity. Cell. (2022) 185:1588–601 e14. doi: 10.1016/j.cell.2022.03.018 35413241 PMC8926873

[91] Goldberg Y Mandel M Bar-On YM Bodenheimer O Freedman LS Ash N. Protection and waning of natural and hybrid immunity to SARS-coV-2. N Engl J Med. (2022) 386:2201–12. doi: 10.1056/NEJMoa2118946 PMC9165562 35613036

[92] Ebinger JE Fert-Bober J Printsev I Wu M Sun N Prostko JC. Antibody responses to the BNT162b2 mRNA vaccine in individuals previously infected with SARS-CoV-2. Nat Med. (2021) 27:981–4. doi: 10.1038/s41591-021-01325-6 PMC8205849 33795870

[93] Yoon S-W Widyasari K Jang J Lee S Kang T Kim S. Kinetics of adaptive immune responses after administering mRNA-Based COVID-19 vaccination in individuals with and without prior SARS-CoV-2 infections. BMC Infect Dis. (2023) 23:732. doi: 10.1186/s12879-023-08728-5 37891503 PMC10604405

[94] Kaplonek P Deng Y Shih-Lu-Lee J Zar HJ Zavadska D Johnson M. Hybrid immunity expands the functional humoral footprint of both mRNA and vector-based SARS-CoV-2 vaccines. Cell Rep Med. (2023) 4:101048. doi: 10.1016/j.xcrm.2023.101048 37182520 PMC10126214

[95] Mitsi E Diniz MO Reine J Collins AM Robinson RE Hyder-Wright A. Respiratory mucosal immune memory to SARS-CoV-2 after infection and vaccination. Nat Commun. (2023) 14:6815. doi: 10.1038/s41467-023-42433-w 37884506 PMC10603102

[96] Sette A Crotty S. Immunological memory to SARS-CoV-2 infection and COVID-19 vaccines. Immunol Rev. (2022) 310:27–46. doi: 10.1111/imr.v310.1 35733376 PMC9349657

[97] Bowman KA Stein D Shin S Ferbas KG Tobin NH Mann C. Hybrid immunity shifts the fc-effector quality of SARS-coV-2 mRNA vaccine-induced immunity. mBio. (2022) 13:e0164722. doi: 10.1128/mbio.01647-22 36000735 PMC9600672

[98] Goel RR Painter MM Apostolidis SA Mathew D Meng W Rosenfeld AM. mRNA vaccines induce durable immune memory to SARS-CoV-2 and variants of concern. Science. (2021) 374:abm0829. doi: 10.1126/science.abm0829 34648302 PMC9284784

[99] Bates TA McBride SK Leier HC Guzman G Lyski ZL Schoen D. Vaccination before or after SARS-CoV-2 infection leads to robust humoral response and antibodies that effectively neutralize variants. Sci Immunol. (2022) 7:eabn8014. doi: 10.1126/sciimmunol.abn8014 35076258 PMC8939472

[100] Bekliz M Adea K Vetter P Eberhardt CS Hosszu-Fellous K Vu DL. Neutralization capacity of antibodies elicited through homologous or heterologous infection or vaccination against SARS-CoV-2 VOCs. Nat Commun. (2022) 13:3840. doi: 10.1038/s41467-022-31556-1 35787633 PMC9253337

[101] Stamatatos L Czartoski J Wan YH Homad LJ Rubin V Glantz H. mRNA vaccination boosts cross-variant neutralizing antibodies elicited by SARS-CoV-2 infection. Science. (2021) 372:1413–8. doi: 10.1126/science.abg9175 PMC8139425 33766944

[102] Our World in Data. Available online at: https://ourworldindata.org/grapher/covid-variants-bar (Accessed October 30, 2024).

[103] Andre M Lau LS Pokharel MD Ramelow J Owens F Souchak J. From alpha to omicron: how different variants of concern of the SARS-coronavirus-2 impacted the world. Biol (Basel). (2023) 12. doi: 10.3390/biology12091267 PMC10525159 37759666

[104] Hirabara SM Serdan TDA Gorjao R Masi LN Pithon-Curi TC Covas DT. SARS-COV-2 variants: differences and potential of immune evasion. Front Cell Infect Microbiol. (2021) 11:781429. doi: 10.3389/fcimb.2021.781429 35118007 PMC8805732

[105] Andrews N Stowe J Kirsebom F Toffa S Rickeard T Gallagher E. Covid-19 vaccine effectiveness against the omicron (B.1.1.529) Variant. N Engl J Med. (2022) 386:1532–46. doi: 10.1056/NEJMoa2119451 PMC8908811 35249272

[106] CEPR. Available online at: https://cepr.org/voxeu/columns/impact-global-covid-19-vaccination-campaign-all-cause-mortality (Accessed October 30, 2024).

[107] Andreano E Paciello I Piccini G Manganaro N Pileri P Hyseni I. Hybrid immunity improves B cells and antibodies against SARS-CoV-2 variants. Nature. (2021) 600:530–5. doi: 10.1038/s41586-021-04117-7 PMC8674140 34670266

[108] Bellusci L Grubbs G Zahra FT Forgacs D Golding H Ross TM. Antibody affinity and cross-variant neutralization of SARS-CoV-2 Omicron BA.1, BA.2 and BA.3 following third mRNA vaccination. Nat Commun. (2022) 13:4617. doi: 10.1038/s41467-022-32298-w 35941152 PMC9358642

[109] Behrens GMN Barros-Martins J Cossmann A Ramos GM Stankov MV Odak I. BNT162b2-boosted immune responses six months after heterologous or homologous ChAdOx1nCoV-19/BNT162b2 vaccination against COVID-19. Nat Commun. (2022) 13:4872. doi: 10.1038/s41467-022-32527-2 35982040 PMC9387891

[110] Edara VV Manning KE Ellis M Lai L Moore KM Foster SL. mRNA-1273 and BNT162b2 mRNA vaccines have reduced neutralizing activity against the SARS-CoV-2 omicron variant. Cell Rep Med. (2022) 3:100529. doi: 10.1016/j.xcrm.2022.100529 35233550 PMC8784612

[111] Muik A Lui BG Wallisch AK Bacher M Muhl J Reinholz J. Neutralization of SARS-CoV-2 Omicron by BNT162b2 mRNA vaccine-elicited human sera. Science. (2022) 375:678–80. doi: 10.1126/science.abn7591 PMC9836206 35040667

[112] Kotaki R Adachi Y Moriyama S Onodera T Fukushi S Nagakura T. SARS-CoV-2 Omicron-neutralizing memory B cells are elicited by two doses of BNT162b2 mRNA vaccine. Sci Immunol. (2022) 7:eabn8590. doi: 10.1126/sciimmunol.abn8590 35113654 PMC8939773

[113] Kim W Zhou JQ Horvath SC Schmitz AJ Sturtz AJ Lei T. Germinal centre-driven maturation of B cell response to mRNA vaccination. Nature. (2022) 604:141–5. doi: 10.1038/s41586-022-04527-1 PMC9204750 35168246

[114] Andreano E Paciello I Pierleoni G Piccini G Abbiento V Antonelli G. B cell analyses after SARS-CoV-2 mRNA third vaccination reveals a hybrid immunity like antibody response. Nat Commun. (2023) 14:53. doi: 10.1038/s41467-022-35781-6 36599850 PMC9811867

[115] Kotaki R Moriyama S Oishi S Onodera T Adachi Y Sasaki E. Repeated Omicron exposures redirect SARS-CoV-2-specific memory B cell evolution toward the latest variants. Sci Transl Med. (2024) 16:eadp9927. doi: 10.1126/scitranslmed.adp9927 39167666

[116] Moss P. The T cell immune response against SARS-CoV-2. Nat Immunol. (2022) 23:186–93. doi: 10.1038/s41590-021-01122-w 35105982

[117] Muller TR Gao Y Wu J Ribeiro O Chen P Bergman P. Memory T cells effectively recognize the SARS-CoV-2 hypermutated BA.2.86 variant. Cell Host Microbe. (2024) 32:156–61 e3. doi: 10.1016/j.chom.2023.12.010 38211584

[118] Head JR Collender PA Leon TM White LA Sud SR Camponuri SK. COVID-19 vaccination and incidence of pediatric SARS-coV-2 infection and hospitalization. JAMA Netw Open. (2024) 7:e247822. doi: 10.1001/jamanetworkopen.2024.7822 38652476 PMC11040406

[119] Comirnaty. Summary of Product Characteristics . Available online at: https://www.ema.europa.eu/en/documents/product-information/comirnaty-epar-product-information_en.pdf (Accessed November 4, 2024).

[120] Spikevax. Summary of Product Characterstics . Available online at: https://www.ema.europa.eu/en/documents/product-information/spikevax-epar-product-information_en.pdf (Accessed November 4, 2024).

[121] Amodio E Genovese D Mazzeo L Martino L Restivo V Vella G. Effectiveness of mRNA COVID-19 vaccines in adolescents over 6 months. Pediatrics. (2022) 150. doi: 10.1542/peds.2022-057394 35945678

[122] Lan Z Yan J Yang Y Tang Z Guo X Wu Z. Effectiveness of COVID-19 vaccines among children and adolescents against SARS-CoV-2 variants: a meta-analysis. Eur J Pediatr. (2023) 182:5235–44. doi: 10.1007/s00431-023-05216-5 37768334

[123] Price AM Olson SM Newhams MM Halasa NB Boom JA Sahni LC. BNT162b2 protection against the omicron variant in children and adolescents. N Engl J Med. (2022) 386:1899–909. doi: 10.1056/NEJMoa2202826 PMC9006785 35353976

[124] Bartsch YC Chen JW Kang J Burns MD St Denis KJ Sheehan ML. BNT162b2 induces robust cross-variant SARS-CoV-2 immunity in children. NPJ Vaccines. (2022) 7:158. doi: 10.1038/s41541-022-00575-w 36463314 PMC9719544

[125] Zhong Y Kang AYH Tay CJX Li HE Elyana N Tan CW. Correlates of protection against symptomatic SARS-CoV-2 in vaccinated children. Nat Med. (2024) 30:1373–83. doi: 10.1038/s41591-024-02962-3 PMC11164684 38689059

[126] Cinicola BL Piano Mortari E Zicari AM Agrati C Bordoni V Albano C. The BNT162b2 vaccine induces humoral and cellular immune memory to SARS-CoV-2 Wuhan strain and the Omicron variant in children 5 to 11 years of age. Front Immunol. (2022) 13:1094727. doi: 10.3389/fimmu.2022.1094727 36591287 PMC9797965

[127] Nziza N Deng Y Wood L Dhanoa N Dulit-Greenberg N Chen T. Humoral profiles of toddlers and young children following SARS-CoV-2 mRNA vaccination. Nat Commun. (2024) 15:905. doi: 10.1038/s41467-024-45181-7 38291080 PMC10827750

[128] Bartsch YC St Denis KJ Kaplonek P Kang J Lam EC Burns MD. SARS-CoV-2 mRNA vaccination elicits robust antibody responses in children. Sci Transl Med. (2022) 14:eabn9237. doi: 10.1126/scitranslmed.abn9237 35881018 PMC9348753

[129] Vaxzevria. Summary of Product Characteristics . Available online at: https://www.ema.europa.eu/en/documents/product-information/vaxzevria-epar-product-information_en.pdf (Accessed Novemeber 7, 2024).

[130] Jcovden. Available online at: https://www.ema.europa.eu/en/documents/all-authorised-presentations/jcovden-previously-covid-19-vaccine-janssen-epar-all-authorised-presentations_en.pdf (Accessed November 24, 2024).

[131] Bimervax. Summary of Product Characteristics . Available online at: https://www.ema.europa.eu/en/documents/product-information/bimervax-epar-product-information_en.pdf (Accessed November 4, 2024).

[132] Nuvaxovid. Summary of Product Chractertistics . Available online at: https://www.ema.europa.eu/en/documents/product-information/nuvaxovid-epar-product-information_en.pdf (Accessed November 4, 2024).

[133] EMA. Available online at: https://www.ema.europa.eu/en/news/ema-recommends-authorisation-nuvaxovid-adolescents-aged-12-17 (Accessed February 12 2025).

[134] Lang K. What do we know about covid in immunocompromised people? BMJ. (2023) 383:1612. 37879752 10.1136/bmj.p1612

[135] Martinson ML Lapham J. Prevalence of immunosuppression among US adults. JAMA. (2024) 331:880–2. doi: 10.1001/jama.2023.28019 PMC10870224 38358771

[136] Evans RA Dube S Lu Y Yates M Arnetorp S Barnes E. Impact of COVID-19 on immunocompromised populations during the Omicron era: insights from the observational population-based INFORM study. Lancet Reg Health Eur. (2023) 35:100747. doi: 10.1016/j.lanepe.2023.100747 38115964 PMC10730312

[137] CDC. Available online at: https://www.cdc.gov/respiratory-viruses/risk-factors/older-adults.html (Accessed October 30, 2024).

[138] Shao L Lie AK Zhang Y Wong CH Kwong YL. Aberrant germinal center formation, follicular T-helper cells, and germinal center B-cells were involved in chronic graft-versus-host disease. Ann Hematol. (2015) 94:1493–504. doi: 10.1007/s00277-015-2394-z 25963280

[139] Bartleson JM Radenkovic D Covarrubias AJ Furman D Winer DA Verdin E. SARS-coV-2, COVID-19 and the ageing immune system. Nat Aging. (2021) 1:769–82. doi: 10.1038/s43587-021-00114-7 PMC8570568 34746804

[140] Collier DA Ferreira I Kotagiri P Datir RP Lim EY Touizer E. Age-related immune response heterogeneity to SARS-CoV-2 vaccine BNT162b2. Nature. (2021) 596:417–22. doi: 10.1038/s41586-021-03739-1 PMC8373615 34192737

[141] Dallan B Proietto D De Laurentis M Gallerani E Martino M Ghisellini S. Age differentially impacts adaptive immune responses induced by adenoviral versus mRNA vaccines against COVID-19. Nat Aging. (2024) 4:1121–36. doi: 10.1038/s43587-024-00644-w 38918602

[142] Vitalle J Perez-Gomez A Ostos FJ Gasca-Capote C Jimenez-Leon MR Bachiller S. Immune defects associated with lower SARS-CoV-2 BNT162b2 mRNA vaccine response in aged people. JCI Insight. (2022) 7. doi: 10.1172/jci.insight.161045 PMC9536264 35943812

[143] Delmonte OM Castagnoli R Notarangelo LD. COVID-19 and inborn errors of immunity. Physiol (Bethesda). (2022) 37. doi: 10.1152/physiol.00016.2022 PMC9550578 35944006

[144] Piano Mortari E Pulvirenti F. COVID-19 infection and vaccination in immunodeficiency. Clin Exp Immunol. (2022) 209:259–61. doi: 10.1093/cei/uxac080 PMC9384801 35972956

[145] Piano Mortari E Pulvirenti F Marcellini V Terreri S Salinas AF Ferrari S. Functional CVIDs phenotype clusters identified by the integration of immune parameters after BNT162b2 boosters. Front Immunol. (2023) 14:1194225. doi: 10.3389/fimmu.2023.1194225 37304298 PMC10248522

[146] Gaete-Argel A Saavedra-Alarcon V Saure D Alonso-Palomares L Acevedo ML Alarcon M. Impact of homologous and heterologous boosters in neutralizing antibodies titers against SARS-CoV-2 Omicron in solid-organ transplant recipients. Front Immunol. (2023) 14:1135478. doi: 10.3389/fimmu.2023.1135478 36999018 PMC10044136

[147] Pulvirenti F Di Cecca S Sinibaldi M Piano Mortari E Terreri S Albano C. T-cell defects associated to lack of spike-specific antibodies after BNT162b2 full immunization followed by a booster dose in patients with common variable immune deficiencies. Cells. (2022) 11. doi: 10.3390/cells11121918 PMC9221747 35741048

[148] Carsetti R Agrati C Pinto VM Gianesin B Gamberini R Fortini M. Premature aging of the immune system affects the response to SARS-CoV-2 mRNA vaccine in beta-thalassemia: role of an additional dose. Blood. (2022) 140:1735–8. doi: 10.1182/blood.2022017594 PMC9420073 36004936

[149] Pulvirenti F Fernandez Salinas A Milito C Terreri S Piano Mortari E Quintarelli C. B cell response induced by SARS-coV-2 infection is boosted by the BNT162b2 vaccine in primary antibody deficiencies. Cells. (2021) 10. doi: 10.3390/cells10112915 PMC8616496 34831138

[150] Bordoni V Casale M Pinto VM Carsetti R Gianesin B Gamberini MR. Inflammatory and senescence-associated mediators affect the persistence of humoral response to COVID-19 mRNA vaccination in transfusion-dependent beta-thalassemic patients. Am J Hematol. (2023) 98:E145–E7. doi: 10.1002/ajh.26905 36871203

[151] Corradini P Agrati C Apolone G Mantovani A Giannarelli D Marasco V. Humoral and T-cell immune response after 3 doses of messenger RNA severe acute respiratory syndrome coronavirus 2 vaccines in fragile patients: The Italian VAX4FRAIL study. Clin Infect Dis. (2023) 76:e426–e38. doi: 10.1093/cid/ciac404 PMC9213871 35607769

[152] Rescigno M Agrati C Salvarani C Giannarelli D Costantini M Mantovani A. Neutralizing antibodies to Omicron after the fourth SARS-CoV-2 mRNA vaccine dose in immunocompromised patients highlight the need of additional boosters. Front Immunol. (2023) 14:1104124. doi: 10.3389/fimmu.2023.1104124 36776853 PMC9911671

[153] CDC. Available online at: https://www.cdc.gov/mmwr/volumes/73/wr/mm7349a2.htm?s_cid=mm7349a2_w (Accessed February 12 2025).

[154] Pardo I Maezato AM Callado GY Gutfreund MC Hsieh MK Lin V. Effectiveness of heterologous and homologous COVID-19 vaccination among immunocompromised individuals: a systematic literature review and meta-analysis. Antimicrob Steward Healthc Epidemiol. (2024) 4:e152. doi: 10.1017/ash.2024.369 39346662 PMC11427957

[155] CDC. Available online at: https://wwwnc.cdc.gov/eid/article/29/2/22-1422_article (Accessed February 12 2025).

[156] Lasrado N Rowe M McMahan K Hachmann NP Miller J Jacob-Dolan C. SARS-CoV-2 XBB.1.5 mRNA booster vaccination elicits limited mucosal immunity. Sci Transl Med. (2024) 16:eadp8920. doi: 10.1126/scitranslmed.adp8920 39441905 PMC11542980

[157] Allie SR Bradley JE Mudunuru U Schultz MD Graf BA Lund FE. The establishment of resident memory B cells in the lung requires local antigen encounter. Nat Immunol. (2019) 20:97–108. doi: 10.1038/s41590-018-0260-6 30510223 PMC6392030

[158] Allie SR Randall TD. Resident memory B cells. Viral Immunol. (2020) 33:282–93. doi: 10.1089/vim.2019.0141 PMC7247033 32023188

[159] Davis-Porada J George AB Lam N Caron DP Gray JI Huang J. Maintenance and functional regulation of immune memory to COVID-19 vaccines in tissues. Immunity. (2024) 57:2895–913 e8. doi: 10.1016/j.immuni.2024.10.003 39510068 PMC11634668

[160] Lim JME Tan AT Le Bert N Hang SK Low JGH Bertoletti A. SARS-CoV-2 breakthrough infection in vaccinees induces virus-specific nasal-resident CD8+ and CD4+ T cells of broad specificity. J Exp Med. (2022) 219. doi: 10.1084/jem.20220780 PMC9386509 35972472

[161] Oh JE Song E Moriyama M Wong P Zhang S Jiang R. Intranasal priming induces local lung-resident B cell populations that secrete protective mucosal antiviral IgA. Sci Immunol. (2021) 6:eabj5129. doi: 10.1126/sciimmunol.abj5129 34890255 PMC8762609

[162] Ramirez SI Faraji F Hills LB Lopez PG Goodwin B Stacey HD. Immunological memory diversity in the human upper airway. Nature. (2024) 632:630–6. doi: 10.1038/s41586-024-07748-8 PMC11895801 39085605

[163] Lavelle EC Ward RW. Mucosal vaccines - fortifying the frontiers. Nat Rev Immunol. (2022) 22:236–50. doi: 10.1038/s41577-021-00583-2 PMC8312369 34312520

[164] Evans JP Liu SL. Challenges and prospects in developing future SARS-coV-2 vaccines: overcoming original antigenic sin and inducing broadly neutralizing antibodies. J Immunol. (2023) 211:1459–67. doi: 10.4049/jimmunol.2300315 37931210

[165] Callaway E. The next generation of coronavirus vaccines: a graphical guide. Nature. (2023) 614:22–5. doi: 10.1038/d41586-023-00220-z 36726000

[166] VidPrevtyn Beta. Summary of Product Characteristics . Available online at: https://www.ema.europa.eu/en/documents/product-information/vidprevtyn-beta-epar-product-information_en.pdf (Accessed November 24, 2024).

[167] EMA. Available online at: https://www.ema.europa.eu/en/human-regulatory-overview/public-health-threats/coronavirus-disease-covid-19/covid-19-public-health-emergency-international-concern-2020-23/withdrawn-applications-products (Accessed November 24, 2024).

[168] Scarpa F Sanna D Benvenuto D Borsetti A Azzena I Casu M. Genetic and structural data on the SARS-coV-2 omicron BQ.1 variant reveal its low potential for epidemiological expansion. Int J Mol Sci. (2022) 23. doi: 10.3390/ijms232315264 PMC9739521 36499592

[169] ECDC. Available online at: https://www.ecdc.europa.eu/en/news-events/update-sars-cov-2-variants-ecdc-assessment-xbb15-sub-lineage:~:text=Disclaimer-,XBB.,are%20associated%20with%20significant%20uncertainty (Accessed February 12 2025).

[170] ICRRS. Available online at: https://www.marionegri.it/magazine/covid-19-varianti-eg-5-e-ba-2-86 (Accessed February 12 2025).

[171] Mohapatra RK Simhachalam Kutikuppala LV Mishra S Sarangi AK Sena Tuglo L. WHO-classified COVID-19 ‘variant of interest’ JN.1: A community health concern in the winter and festive season. Clin Infect Pract. (2024) 21:100348. doi: 10.1016/j.clinpr.2024.100348

[172] Kaku Y Uriu K Kosugi Y Okumura K Yamasoba D Uwamino Y. Virological characteristics of the SARS-coV-2 KP.2 variant. Lancet Infect Dis. (2024) 24:e416. doi: 10.1016/S1473-3099(24)00298-6 38782005

[173] Rajput V Das R Pramanik R Nannaware K Sushma Y Taji N. Early detection of KP.2 SARS-coV-2 variant using wastewater-based genomic surveillance in Pune, Maharashtra, India. J Travel Med. (2024). doi: 10.1093/jtm/taae097 39018237

[174] ECDC. Available online at: https://www.ecdc.europa.eu/en/covid-19/variants-concernvariants-of-concern-voc (Accessed February 12 2025).

